# Volume and its relationship to cardiac output and venous return

**DOI:** 10.1186/s13054-016-1438-7

**Published:** 2016-09-10

**Authors:** S. Magder

**Affiliations:** Department of Critical Care, McGill University Health Centre, 1001 Decarie Blvd., Montreal, Quebec H4A 3J1 Canada

**Keywords:** Cardiac output, Venous return, Compliance, Capacitance, Circulatory filling pressure, Mean systemic filling pressure, Time constants, Stressed volume

## Abstract

Volume infusions are one of the commonest clinical interventions in critically ill patients yet the relationship of volume to cardiac output is not well understood. Blood volume has a stressed and unstressed component but only the stressed component determines flow. It is usually about 30 % of total volume. Stressed volume is relatively constant under steady state conditions. It creates an elastic recoil pressure that is an important factor in the generation of blood flow. The heart creates circulatory flow by lowering the right atrial pressure and allowing the recoil pressure in veins and venules to drain blood back to the heart. The heart then puts the volume back into the systemic circulation so that stroke return equals stroke volume. The heart cannot pump out more volume than comes back. Changes in cardiac output without changes in stressed volume occur because of changes in arterial and venous resistances which redistribute blood volume and change pressure gradients throughout the vasculature. Stressed volume also can be increased by decreasing vascular capacitance, which means recruiting unstressed volume into stressed volume. This is the equivalent of an auto-transfusion. It is worth noting that during exercise in normal young males, cardiac output can increase five-fold with only small changes in stressed blood volume. The mechanical characteristics of the cardiac chambers and the circulation thus ultimately determine the relationship between volume and cardiac output and are the subject of this review.

## Background

Ernest Starling [1] recognized at the turn of the last century that the heart can only pump out what comes back to it. This concept was later developed further by Arthur Guyton [2] and Solbert Permutt [3, 4]. The significance of this concept is the implication that the mechanical characteristics of the circulation are major determinants of cardiac output. The key mechanical terms that need to be understood are compliance, capacitance, stressed volume, and resistance to flow. This review begins with definitions of these terms. Next, the determinants of flow are presented in a simple model that lumps all compliances of the circuit in one region into what is called a lumped parameter model. More complex models are then discussed. These include the compliant regions that exist between the two ventricles in pulmonary vessels and a model, known as the Krogh model [5], in which the systemic circulation has two systemic venous compliant regions in parallel, one with a high compliance and the other with a low compliance. Lastly, the implications of the mechanics of the circulation for clinical use of fluids and drugs to support the circulation is discussed. These concepts have been reviewed previously [6, 7] but in this paper the emphasis will be on the role of volume as a determinant of cardiac output because so much of the management of critically ill patients revolves around infusions of volume.

## Constant volume

A central axiom in the circulation is that vascular volume is constant under steady state conditions. This volume stretches the elastic walls of the vasculature structure and creates an elastic recoil force that is present even when there is no flow but is also a key determinant of flow [4, 8]. The potential energy of this elastic recoil becomes evident when there is no flow in the circulation and large veins are opened to atmospheric pressure. Vascular volume empties from the veins even without cardiac contractions. The heart adds a pulsatile component to this static potential energy which redistributes the volume according to the compliances and resistances entering and draining each elastic compartment of the circulation.

It may sound obvious that vascular volume is constant but this point is often neglected. A key example is the use of electrical circuitry to model circulatory flow. Electrical models are based on Ohm’s law, which says that the difference in charge in volts (V) equals the product of the current (I), which is the amount of electrons per time, and resistance (R), which is the energy loss due to the flow of electrons through the conducting substance. In hydrodynamics, the study of flowing liquids such as in the circulation, pressure is the equivalent of voltage, cardiac output in liters per minute is the equivalent of current, and resistance is the frictional loss of energy due to the interactions of the layers of the moving fluid with the vessel wall. In electrical models voltage is determined by the decrease in charge from a fixed source, such as a wall socket or battery to a ground value. An increase in resistance or change in voltage across the system changes the amount of electrons in the system. In the vasculature this change in number of electrons is the equivalent of a change in volume. In contrast to an electrical system, the vascular volume is constant in the circulation and the pressure drop, the equivalent of the voltage gradient, changes with changes in resistance or volume. The output from the heart shifts volume to the arterial compartment and creates an arterial pressure which is dependent upon the total vascular resistance. It is thus the volume per time, i.e. cardiac output coming out of the heart, that determines arterial pressure rather than the arterial pressure determining the volume per time or flow in the system.

## Compliance

**Fig. 1:**
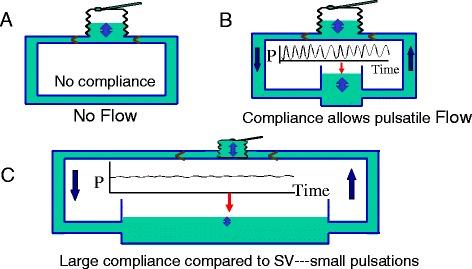
The importance of a compliant region in the circulation. **a** A bellows trying to pump fluid around a system with stiff pipes and no compliance. Flow is not possible because pressing on the bellows instantly raises the pressure everywhere and there is no pressure gradient for flow. **b** An open compliant region which allows changes in volume for changes in pressure. Flow can occur and there are pulsations throughout. **c** The compliant region is much large than in (**b**). The pulsations are markedly dampened and only produce ripples on the surface of the compliant region

Compliance is a measure of the distensibility of a spherical structure and is determined by the change in volume for a change in pressure. A simple example is inflation of a balloon with a known volume and then measuring the change in pressure across the wall. It may seem surprising that this static property is a key determinant of flow, which is a dynamic state. The importance of compliance is that the elastic recoil force created by stretching the walls of vascular structures creates a potential force that can drive flow when the downstream pressure is lower. Second, compliance is necessary to allow pulsatile flow through a closed circuit (Fig. 1). Cardiac contractions create a volume wave that moves through the vasculature. The walls of vessels must be able to stretch in order to transiently take up the volume. The pressure created by the stretch of vascular walls moves the volume on to the next vascular segment with a lower pressure. If vascular walls were all very stiff, pressure generated by a pump at one end would be instantaneously transmitted throughout the vasculature. The pressure would then be equal at the start and end of the circuit and there would be no pressure gradient for flow.

The sum of compliances in a series is the sum of the compliances of all the parts. The compliance of small venules and veins is almost 40 times greater than that of arterial vessels [9, 10] and large veins; capillaries have an even smaller compliance; and the compliance of the pulmonary arterial and venous compartments is about one-seventh that of the systemic circulation [11]. Thus, the total compliance of the circulation is dominated by the compliance of the systemic veins and venules, which contain over 70 % of total blood volume at low pressure. Because most of the compliance resides in this one region, for a first approximation the circulation can be considered as having one compliance lumped in the veins and venules. This simplification creates an approximate 10 % error in the prediction of changes in flow with changes in volume but it makes the mathematics much simpler. The implications of this simplification will be discussed later. Of note, if the question is what determines arterial pulse pressure rather than cardiac output, the small arterial compliance is the key value to consider and total vascular compliance is not important.

As already discussed, when the vasculature is filled with a normal blood volume but there is no flow, the vasculature still has a pressure and this pressure is the same in all compartments of the circulation. It is called mean circulatory filling pressure (MCFP) and is determined by the total stressed volume in the circulation and the sum of the compliances of all regions, including the pulmonary and cardiac compartments [12]. By reducing the volume in the right heart and lowering diastolic pressure, the beating heart allows the elastic recoil pressure in veins and venules to drain back to the heart. The heart thus acts in a “permissive” role by allowing the recoil force that is already present in the veins and venules to act. The heart also has a “restorative” role in that it puts the blood that drained from the veins and venules back into the systemic compartment. With each beat in the steady state, a stroke return is removed from the vena cava to refill the right ventricle and an equal amount of volume is added back to the arterial side as stroke volume. On the venous side stroke return can continue during the whole cycle because atria take up volume even when the ventricles are injecting, but on the arterial side the stroke volume only occurs during systole. The stroke volume is all the volume that moves through the system on each beat.

Some argue that instead of the heart merely being permissive and just allowing venous recoil, flow through the circulation occurs because the volume pumped out by the heart creates an increase in arterial pressure that drives the flow through the system and even determines the right atrial pressure [13, 14]. However, this type of reasoning ignores a number of issues. Flow occurs from areas of high pressure to areas of lower pressure. The flow back to the heart occurs from the upstream veins and venules. The pressure in this region is determined by the volume these vessels contain and the compliance of their walls. As already noted this region contains the bulk of circulating volume and the heart has little volume that it can add to the veins and venules and cannot significantly increase the pressure in these vessels. Thus, the pressure in this region remains relatively constant and flow occurs by lowering the downstream right atrial pressure through the actions of the right heart rather than by increasing the upstream pressure. If the heart rate were limited to one beat per minute, the pressure in the system would equilibrate in all regions before the next beat. The arterial pulse pressure is created by the single stroke volume ejected by the heart and the resistance to its drainage from the arterial compartment and the volume remaining in the aorta at the end of diastole. Widely different stroke volumes can be observed with the same arterial pressure, depending upon the arterial resistance. For example, in sepsis the cardiac output is high and blood pressure is low. During exercise cardiac output can increase more than fivefold with little change in mean arterial pressure [15].

A useful analogy for understanding the importance of the large compliance in veins and venules, and why the pressure produced by the heart is not important for the return of blood, is that of a bathtub [16]. The rate of emptying of a bathtub is dependent upon the height of water above the opening at the bottom of the tub. The height of water creates a hydrostatic pressure due to the mass of the water and the force of gravity on its mass, which pushes the water through the resistance draining the tub. However, the flow out of the tub is not affected by the pressure coming out of the tap. The tap can only alter the outflow from the drain by adding volume and increasing the height of water in the tub. Only the volume flowing into the tub per minute is important for outflow and not the inflow pressure. Over short time periods the flow from the tap has very little effect on the height of water because the surface of the tub is very large compared with the height of water; that is, the tub is very compliant. The same is true in the circulation. Arterial pressure flowing into veins and venules does not affect the flow out of the veins. As in the bathtub, only the liters per minute flowing from the arteries into the veins affects how the veins and venules empty. Furthermore, a bathtub has an unlimited upstream source of volume that can be added to it but in the circulation there is very little other volume that the heart can add to the veins and venules to change their recoil pressure because they already contain the bulk of vascular volume. To take the analogy further, if the bathtub is filled to the brim, any additional volume just flows over the edge of the bathtub and does not change flow out of the drain. The equivalent of this in the body is what occurs when veins and venules are overfilled, whether by clinical intervention or retention of volume through renal mechanisms. The increase in venous pressure increases leakage from the upstream capillaries into the interstitial space and is like spilling the volume on the floor with only minimal changes in venous return.

If a sink were considered instead of a bathtub, the inflow from the tap would have a much greater effect on outflow because the sink is effectively much less compliant than a bathtub. A much smaller change in volume is needed to increase the height of water in the sink and therefore the outflow. Later, I will discuss the significance of having the equivalence of a bathtub and sink in parallel and the potential to change the distribution of flow going to each of them in what is called the Krogh model, which was first described in 1912 [5].

## Capacitance

In electrical models capacitance is the equivalent of compliance but in hydraulic models capacitance means something very different [17–21]. Elastic structures have a resting length that is not stretched. In vessels, too, a volume is needed for the structure to be round but only the volume beyond this resting length produces tension in elastic walls of vascular structures. The volume that stretches the walls is called stressed volume and the rest is called unstressed volume [19, 22]. In the circulation with minimal sympathetic tone, 25 to 30 % of total blood volume is stressed and the rest is unstressed. Thus, in someone with a total blood volume of 5.5 L, only about 1.3 to 1.4 L of blood actually stretches the walls and produces the recoil force. Capacitance is the total contained volume that can be contained at a given pressure and includes unstressed and stressed volume. Importantly, capacitance can be changed by contraction or relaxation of vascular smooth muscles. A decrease in capacitance occurs when vascular smooth muscles of veins and venules shorten. This recruits unstressed into stressed volume so that total volume is at a higher pressure [17]. With a normally filled vasculature in a 70 kg male, extreme sympathetic activation can recruit as much as 18 ml/kg of unstressed into stressed volume [18, 19, 23–25]. A more moderate 10 ml/kg recruitment would expand stressed volume by 700 ml and almost double the stressed volume. This increase in stressed volume occurs in seconds for it is a reflex process. Removal of sympathetic drive can equally remove this equivalent amount of stressed volume in seconds and lead to a marked fall in MCFP [9, 26–28]. This is called an increase in vascular capacitance. A de-recruitment of volume of 10 ml/kg in this example would decrease MCFP by half. The importance of this for the regulation of cardiac output will be discussed later.

## Resistance

Resistance accounts for the frictional loss of energy as blood flows through the vasculature and is calculated from the difference between the inflow pressure and the outflow pressure divided by the flow. The circulation can be considered to be divided into a series of compliant regions that go from one to another through a resistance. Under flow conditions, the pressure in each region is determined by the volume and compliance of that region. The right heart is fed by the large compliant venules and small veins. The pressure in this region is called mean systemic filling pressure (MSFP) instead of MCFP, for it is only related to the volume in the systemic veins. MSFP must equal MCFP under the condition of no flow. Under normal flow conditions they usually are quite similar because the systemic veins dominate total compliance but, depending upon the relative functions of the right and left ventricles and changes in circuit resistances, MSFP can be either higher or lower than MCFP.

**Fig. 2:**
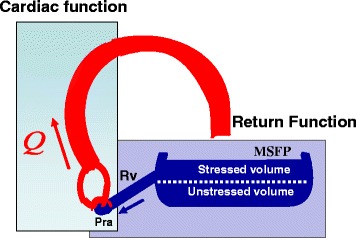
Cardiac output is determined by the interaction of a cardiac function and a return function. *MSFP* is mean systemic filling pressure, *Rv* is the resistance to venous return, and *Pra* is right atrial pressure

**Fig. 3:**
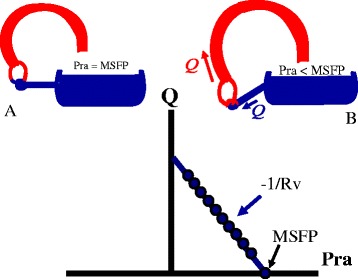
Guyton’s graphical analysis of the return function. **a** When right atrial pressure (*Pra*) equals MSFP, flow in the system is zero. **b** Flow occurs when the cardiac function lowers Pra with a linear relationship between flow and Pra. The slope is minus one over the resistance to venous return (*Rv*)

**Fig. 4:**
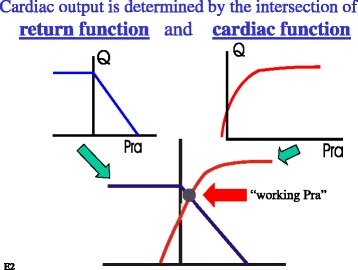
Guyton’s approach to solving the intersection of the return function (venous return curve) and cardiac function curve. Since these two functions have the same axes, they can be plotted on the same graph. Where they intersect gives the working right atrial pressure (*Pra*), cardiac output, and venous return for the two functions

The working cardiac output, venous return, and right atrial pressure are determined by the interaction of the function of the heart, or cardiac function, and the function of venous drainage, i.e., venous return (Fig. 2). Arthur Guyton appreciated that this intersection value can easily be solved graphically by plotting right atrial pressure on the x-axis and flow on the y-axis (Fig. 3) [2, 29]. In a series of animal experiments that allowed separation of the heart and circuit, Guyton and colleagues showed that progressively lowering right atrial pressure produces a linear increase in venous return up to a maximum value, which will be discussed later [29]. The slope of this line is minus one over the resistance draining the veins and the x-intercept is MCFP. A plot of cardiac function on the same graph gives the intersection value of the two functions (Fig. 4). Note that the cardiac function curve starts from a negative value on the graph. This is because when the heart is empty its transmural pressure is equal to the surrounding pressure, which is pleural pressure and not atmospheric pressure. During spontaneous breathing pleural pressure is negative at end-expiration and at functional residual capacity.

## Limits of cardiac and return functions

**Fig. 5:**
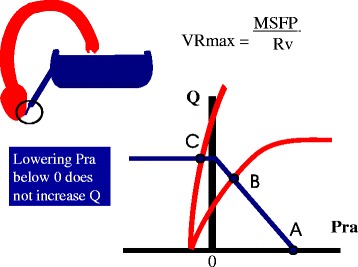
Limit of the return function. When the pressure inside the great veins is less than the surrounding pressure (which is zero when breathing at atmospheric pressure), the vessels collapse and there is flow limitation. Lowering right atrial pressure (*Pra*) further does not increase flow. Maximum venous return (*VRmax*) is then dependent upon MSFP and venous resistance (*Rv*). The heart cannot create a flow higher than this value

**Fig. 6:**
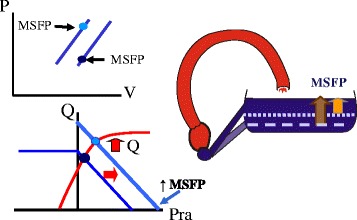
Change in cardiac output and venous return with an increase in capacitance. An increase in capacitance is the same as lowering the opening on the side of a tub for it allows more volume to flow out, which is the equivalent of more volume being stressed. Graphically it results in a leftward shift of the volume–pressure relationship of the vasculature (*upper left*). This shifts the venous return curve to the right and increases cardiac output through the Starling mechanism (*lower left*). This effect is identical to giving volume to expand stressed volume. *Pra* right atrial pressure

Venous return becomes limited when the pressure inside the great veins is less than the pressure outside their walls because the floppy walls of veins collapse and produce what is called a vascular waterfall or a flow limitation (Fig. 5) [30]. This occurs at atmospheric pressure when breathing spontaneously. When venous collapse occurs, further lowering right atrial pressure does not increase venous return [31]. This means that the best the heart can do to increase cardiac output is lower right atrial pressure to zero (Fig. 5). Maximum venous return and cardiac output are defined by the MSFP, which is determined by stressed volume in the veins and venules and the compliance of these structures and by the resistance to venous return. Maximum flow would occur, albeit for an instant, if the heart were suddenly taken out of the circulation, indicating that the heart gets in the way of the return of blood by creating a downstream pressure greater than zero. In patients mechanically ventilated with positive end-expiratory pressure, flow limitation occurs at a positive value and not zero [32]. When venous return is limited, cardiac output can only be increased by increasing MSFP by a volume infusion, by decreasing capacitance and recruiting unstressed into stressed volume without a change in total volume (Fig. 6), or by decreasing the resistance to venous return.

The cardiac function curve, too, has a limit. This occurs because of the limit to cardiac filling due to restraint by the pericardium [33] or, if there is no pericardium, by the cardiac cytoskeleton itself. In pathological situations mediastinal structures can also impose limits on cardiac filling. When cardiac filling is limited, increasing diastolic filling pressure does not increase stroke volume because there is no change in end-diastolic volume and thus no increase in sarcomere length. Under this condition cardiac output can only be increased by an increase in heart rate or cardiac contractility or by a decrease in afterload. When cardiac filling is limited, cardiac output is independent of changes in the venous return function.

## Cardiac contractions and flow of blood

Blood flows down an energy gradient, which generally means that blood flows from an area of high pressure to an area with a lower pressure. As already discussed, pressure in each compartment is determined by the compliance of the wall of the compartment and the volume it contains. As discussed elsewhere in this series, ejection of blood by the heart occurs by what is called a time-varying elastance, which means that the elastance of the walls of the cardiac chambers markedly increase during systole. Conversely, the compliance of the heart markedly decreases during systole. This greatly increases the pressure in the volumes contained in the ventricles. The peak pressure achieved in the ventricle by the cyclic decreases in compliance depends upon where the volume is along the ventricular end-systolic volume–pressure line, the pressure in the aorta at the onset of ventricular contraction, and how easy it is for blood to flow out of the aorta, which is dependent upon the downstream arterial resistance and critical closing pressures [34]. This produces a pulse pressure which is determined by the elastance of the aorta [35]. The increase in aortic volume stretches its walls and creates a pressure gradient from the arteries to the veins. The consequent change in volume in the veins minimally increases the pressure in that region (i.e., MSFP) because it is so compliant (Fig. 1). During diastole the compliance of the right and left heart markedly increase and the pressures in the ventricles markedly fall because there is much less volume than before the systolic decrease in compliance. Since there is little change in MSFP and a marked drop in the downstream right atrial pressure, it is evident that the major factor affecting the return of blood to the heart is the lowering of right atrial pressure by the action of the right heart and not the trivial change in MSFP or the pressure in the aorta.

## Time constants

The rhythmic pulsations produced by the time-varying elastances of the ventricles produce important limitations to blood flow. When a step increase is made in flow to a compliant system with an outflow resistance, the pressure does not increase with a step change but rather rises to the new value exponentially. The rate of rise is determined by what is called a time constant (τ) [3, 4], which is the time it takes to get to 63 % of the new steady state and is determined by the product of compliance and resistance draining the region. This means that if there is not enough time in the cycle to reach the new steady state, volume will be trapped in the upstream compartment. Thus, besides just pressure gradients, heart rate becomes a factor in cardiac filling and emptying. The equation for venous return (VR) is:

1 $$ VR=\frac{MSFP-Pra}{Rv} $$

where MSFP is mean systemic filling pressure, Pra is right atrial pressure, and Rv is venous resistance.

MSFP is determined by stressed volume (γ) divided by venous compliance (Cv). This can be substituted into Eq. 1 and rearranged to give:

2 $$ VR=\frac{\gamma -Pra\times Cv}{RvCv} $$

where Pra is right atrial pressure. Pra × Cv can be considered as a residual volume left in the ventricle. Since τ equals the product of Rv and Cv:

3 $$ VR=\frac{\gamma -Pra\times Cv}{\tau } $$

This indicates that venous return is determined by stressed volume and the time constant of venous drainage. If cardiac filling time is too short for the returning volume to fill the heart in the time available, the upstream volume and thus pressure must increase or τ must be reduced to maintain the same flow. Compliance of vessels does not change, at least over short periods of time, so that τ can only decrease by a decrease in venous resistance. All this assumes that the limit is due to too short a filling time and that there is not already a limit imposed by the steep portion of the diastolic filling curve. During normal circulatory adjustments to high flow needs, as occur during aerobic exercise, reduction in venous resistance and the distribution of flow as described in the Krogh model below are necessary to allow greater rates of venous return. These occur by matching changes in regional resistances to metabolic activity. This co-ordination of resistances does not occur properly in sepsis and could explain why volume needs to be used to increase MSFP in sepsis to allow for a sufficient cardiac output to match the fall in arterial resistance during distributive shock.

## Pulmonary compliance and volume shifts between systemic and pulmonary circuits

So far in this review cardiac function has been considered as one unit starting from the right atrium and exiting from the aortic valve. Pulmonary vessels and independent functions of the right and left ventricle have not been considered. This simplification normally produces a small error because total pulmonary compliance is only one-seventh of total systemic vascular compliance [11] and the pulmonary circuit does not contain a lot of volume that can be shifted to the systemic circulation. It also cannot take up a lot of volume without causing a large increase in pulmonary venous pressure and a major disturbance to pulmonary gas exchange. Even maximal sympathetic stimulation results in only a small shift from the pulmonary circuit to the systemic circulation [36]. However, the small volume reserves in the pulmonary vasculature become important during the variation in pulmonary flow during ventilation, especially during mechanical ventilation and increases in pleural pressure.

The normal gradient for venous return is only in the range of 4 to 8 mmHg. Because the heart is surrounded by pleural pressure and not atmospheric pressure, an increase in pleural pressure of 10 mmHg during a mechanical breath would cut the inflow to the heart to zero and there would be no stroke volume on beats at peak inspiration [37]. One would thus expect marked variations in left-sided output and arterial pressure during mechanical ventilation, but normally they are moderate. This is because the volume in the compliant component of the pulmonary vasculature provides a reservoir that can sustain left heart filling for the few beats that are necessary during the peak inspiratory pressure. This can be called pulmonary buffering [37].

The compliant compartment of the pulmonary circulation is also important under two pathological conditions. When there is a disproportionately greater decrease in left heart function than right heart function, stressed volume accumulates in the pulmonary circuit because higher filling pressures are needed by the left heart to keep up with the output of the more efficient right heart [11, 38]. In modeling studies without reflex adjustments and failure of the right heart, this leads to a rise in pulmonary venous pressure, which is the upstream reservoir for the left ventricle, and a decrease in MSFP [37]. The fall in MSFP reduces venous return and cardiac output. This would be hard to detect in a patient because fluid retention by the kidney, reflex adjustments, or exogenous fluid administration increase total blood volume and restore MSFP and cardiac output. Accumulation of volume in the pulmonary vasculature is especially a problem in patients with marked left ventricular diastolic dysfunction. In these cases the left-sided filling pressure needs to be higher than normal to maintain adequate stroke volume and cardiac output to perfuse vital organs such as the kidney. However, the higher left ventricular filling pressure increases pulmonary capillary filtration and leads to pulmonary edema and respiratory failure. If volume is removed to treat the respiratory failure, cardiac output deceases and the kidneys fail. If volume is then added to improve renal perfusion, respiratory failure occurs. There is no obvious solution to this clinical problem.

A second mechanism that can increase the proportion of vascular volume in the pulmonary compartments is an increase in the proportion of the lung in West zones 1 and 2 [37, 39]. Under these conditions alveolar pressure becomes the downstream pressure for pulmonary flow instead of left atrial pressure. When this happens, pulmonary venous pressure rises one-to-one with an increase in alveolar pressure and provides a considerable load for the right ventricle. The increased pressure is also downstream of pulmonary capillaries and will increase pulmonary capillary filtration.

Another factor that is not often taken into account when considering the distribution of volume and maintenance of normal pressure gradients for venous return is the size of the heart. Typical limits of diastolic volumes of normal ventricles are in the range of 120 to 140 ml, which between the two ventricles can account for as much as 20 % of stressed volume. In someone who has very dilated ventricles this could be an even higher proportion of total blood volume, although presumably most of it is unstressed. Excess accumulation of the volume in the heart is prevented by the characteristics of the passive filling curves of the ventricles, which become very steep at a value appropriate for a normal stroke volume. If the capacity of the ventricles is too large for the volume reserves of the body, accumulation of volume in the ventricles could take up a significant proportion of systemic venous volume, which would decrease MSFP and limit cardiac output.

## Krogh model

**Fig. 7:**
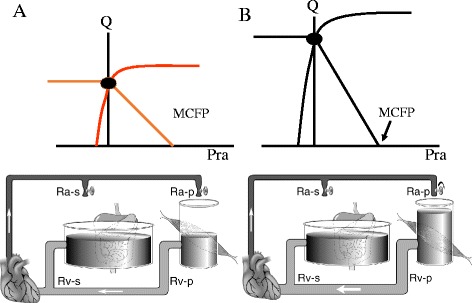
The two-compartment Krogh model. In this model the systemic circulation has a large compliant region (such as the splanchnic vasculature) in parallel with a low-compliance region (equivalent of the peripheral vasculature). A shift in the fractional flow to the low-compliance region by decreasing the arterial resistance (*Ra-p*) into this region decreases venous resistance (upward shift of the slope in **b** compared with **a**) but does not change MCFP. *Rv-s* is splanchnic venous resistance, *Rv-p* is peripheral venous resistance. (Used with permission from reference [5])

So far in this review the systemic vascular compliance has been lumped into one region with a large compliance. Early in the last century August Krogh [5] indicated that if the vasculature consists of an area with a high compliance in parallel with an area with much lower compliance, the fractional distribution of flow between these two regions affects venous return (Fig. 7). This can be understood by the previous discussion on time constants of drainage. If both regions have the same venous resistance, the region with the larger compliance will have a longer time constant of drainage because τ is determined by the product of resistance and compliance [3, 4]. As indicated above, a sink has less compliance than a bathtub, so these two parallel compliances can be considered as a bathtub and sink in parallel. Because of its smaller surface area, a smaller amount of volume is needed to raise the height of water in the sink and to increase the outflow; a sink thus has a fast time constant compared with a bathtub, which has a large surface area and requires a large amount of volume to go through the outflow resistance to change the height of water. Permutt and colleagues demonstrated that the splanchnic vasculature has a time constant of drainage in the range of 20 to 24 s [26, 40, 41], whereas that of the peripheral vasculature bed has a time constant of 4 to 6 s. In this two-compartment model the venous return equation can be written as follows:

4 $$ VR=\frac{\gamma -Pra\times {C}_T}{\;\left({F}_{sp}\times {\tau}_{sp}\right)+\left({F}_{per}\times {\tau}_{per}\right)\;} $$

where C_T_ is total compliance, F_sp_ is the fraction of flow to the splanchnic region, and F_per_ is the fraction of flow to the peripheral regions. τ_sp_ is the product of the splanchnic resistance and compliance and τ_per_ is the product of the peripheral compliance and resistance. Note that if F_per_ were 0 and F_sp_ were 1, this equation is the same as Eq. 3.

The vasculature could be subdivided into smaller regions and, by specifying their drainage characteristics, the analysis could be refined, but use of two groups is sufficient to understand the broad consequences. I will thus simply refer to the splanchnic bed as the high-compliance region and the peripheral region, which is composed primarily of muscle, as the low-compliance regions. These also could be considered as the slow and fast time constant beds, respectively.

Under resting conditions approximately 40 % of blood flow goes to the splanchnic bed and 60 % goes to the peripheral vasculature. Distribution of flow between the two is determined by regional arterial resistances. Importantly, it is the fraction of total cardiac output to each region that is important and not actual flow. This is because total blood volume is constant. A good example of how this functions is the defense against a fall in blood pressure by the baroreceptors [9]. We analyzed this in an animal model in which we controlled the baroreceptor pressures in what is called an open-loop model. This means that the sensor for the perturbation, in this case blood pressure, is separated from the response. We isolated the outflow from the splanchnic and peripheral beds and controlled cardiac output with a pump. This allowed us to assess the venous resistances, compliances, regional flows, and stressed volumes in all compartments. As expected, a decrease in baroreceptor pressure from 200 to 80 mmHg produced a marked rise in arterial resistance. However, the increase in arterial resistance was greater in the peripheral region than the splanchnic region, which redistributed blood flow to the slow time constant splanchnic bed. This makes sense from an evolutionary point of view for it would protect delicate abdominal structures [23], but the consequence of increasing the fraction of flow to the slow time constant bed is a decrease in cardiac output and this would decrease blood pressure further. Other adaptations are thus necessary. The sympathetic output contracted venous vessels in the splanchnic bed and produced a decrease of the venous capacitance of this region, which increased the stressed volume by approximately 10 ml/kg. There was no change in capacitance in peripheral beds for they have a smaller volume reservoir. Strikingly, at the same time that arterial resistance to the splanchnic bed increased, the venous resistance draining this bed decreased. This decreased the time constant of drainage from the splanchnic bed. All together, these adaptations would have increased cardiac output by 110 %. In this two-compartment model the time constants of flow into and out of each region become important because they affect the distribution of flow and emptying of the regions with changes in heart rate and blood pressure and this adds a further complexity to the analysis. These factors are likely important for the responses to vasopressors and inotropic agents. The change in capacitance was an important part of the reflex response but this only can occur if there is adequate unstressed volume to recruit. Unfortunately, unstressed volume cannot be measured in an intact person and thus clinicians must think about the potential unstressed reserves.

## Implications of the physiology for clinical interventions

### Volume therapy

The existence of unstressed volume and the ability to adjust stressed volume by changes in capacitance introduces a role for volume infusions that is not simply to increase cardiac output but rather to ensure reserves. Patients who have had volume losses and whose MSFP is being supported by a reduction in vascular capacitance by recruitment of their unstressed reserves no longer can use this mechanism to rapidly adjust stressed volume as needed. Volume infusion could potentially restore these reserves without producing much change in cardiac output, although there might be some decrease in heart rate because of a decrease in sympathetic activity. However, the response to the next stress would be very different. This argues for infusion of some volume before major surgical interventions and in initial trauma management in subjects who might have reduced volume reserves based on their “volume history”. Note that this would likely not produce much change in any measureable hemodynamic values, including ventilation-induced variations in arterial pressure or stroke volume.

Although use of volume boluses to increase cardiac output is one of the most common clinical interventions in patients in shock, increasing preload is not the major way that the body normally produces large changes in cardiac output [42]. Under normal conditions the Frank–Starling mechanism primarily provides fine adjustment to cardiac function by making sure that the same volume that fills the ventricles on each beat leaves them. For example, during peak aerobic exercise there is very little change in right atrial pressure with the very large increases in cardiac output [43]. The increase in cardiac output occurs by increases in heart rate, contractility, and peripheral mechanical adaptations that allow more venous return. This is not to say that fluids should not be used for resuscitation of patients in shock. Use of fluids can avoid the need for central venous cannulation and the need for drug infusion but it is necessary to understand the limits of what fluids can do. As already discussed, stressed volume normally only is in the range of 20 to 22 ml/kg. In a 70 kg man with a stressed volume of 1400 ml and a MCFP of 10 mmHg, an infusion of a fluid that increased stressed volume by 1 L would increase MCFP to 17 mmHg and likely produce a significant increase in vascular leak. More than likely the liter of fluid would not stay in the vasculature and the effect would be transient. Furthermore, the important MSFP would not rise as much as MCFP because the volume would be distributed in all compartments. If there is left ventricular dysfunction or non-West zone 3 conditions in the lungs, a greater than normal proportion of the fluid would be distributed to the pulmonary compartments [37]. When the two-compartment Krogh model is considered, the effect of the volume becomes even more complicated. The effect of the increase in stressed volume will be much greater if a greater fraction of the blood flow goes to the fast time constant muscle bed because this region is much less compliant and the increase in volume produces a greater increase in the regional elastic recoil pressure. However, this also means that the equivalent of MSFP in the muscle region will be even higher than the estimate given above and be an even greater force for capillary filtration.

### Adrenergic drugs

The study on the effect of the baroreceptor response to hypotension discussed above [9] gives insight into the response of the peripheral circulation to infusions of norepinephrine. Besides the expected increase in systemic arterial resistance, norepinephrine constricts the splanchnic venous compartment and increases stressed volume. It potentially dilates or at least does not constrict the venous drainage from the splanchnic bed. This is because activation of alpha-adrenergic receptors constricts the venous drainage of the splanchnic vasculature whereas beta-adrenergic receptors dilate it [41]. Through its beta-adrenergic activity norepinephrine increases cardiac function and has little effect on pulmonary vessels [44]. The increase in precapillary resistance vessels and the decrease in right atrial pressure with the improvement in cardiac function could potentially decrease capillary filtration and thus could reduce edema formation. However, it is possible that very high levels of norepinephrine compromise the normal distribution of flow and compromise organ function. Epinephrine likely works in the same way [26] except that it generally produces a greater increase in heart rate, which could produce problems by shortening diastole and producing unexpected changes in distribution of flow due to the limits of time constants in different vascular beds, on both the arterial and venous side.

The response of the circuit to phenylephrine is very different from the response to norepinephrine because it only has alpha-adrenergic activity [45, 46]. Although phenylephrine can constrict the splanchnic capacitance vessels, it increases the venous resistance draining this region and the net effect on venous return depends upon how much volume is recruited versus how much the downstream resistance increases. In most critically ill patients capacitance reserves are reduced so that the net effect with phenylephrine is decreased splanchnic drainage and decreased venous return. Phenylephrine also does not increase cardiac function so that cardiac output most often falls [47].

Besides increasing cardiac contractility, an effective inotrope must also alter circuit properties to increase venous return. The circuit properties of dobutamine have not been well studied but we observed in dogs (unpublished data) that dobutamine decreased the resistance draining splanchnic vessels as observed with isoproterenol [41] and also increased MSFP. The latter likely occurred because the d-isomer of dobutamine has alpha-adrenergic activity and thus could constrict capacitance vessels. These circuit adaptations thus combine with dobutamine’s inotropic effects on the heart to increase cardiac output. This also would predict that the effect of dobutamine would be best when there are adequate reserves in unstressed volume to be recruited so that volume infusions could potentially augment its action.

## Conclusions

The circulation starts with a potential energy which is due to the stretching of the elastic walls of all its components by the volume it contains even when there is no blood flow. This volume and the consequent potential energy is constant under steady state conditions but can be changed by recruitment of unstressed volume into stressed volume through what is called a decrease in capacitance, reabsorption of interstitial fluid into the vascular compartment, ingestion and absorption of fluid through the gut, or parenteral fluid administration by health care personnel. A basic principle is that the heart cannot put out more than what it gets back from the large reservoir of volume in the systemic circulation. The time-varying elastance of the ventricles transiently raises the pressure in the volume they contain. This creates a volume and a pressure wave that are dependent upon the downstream resistance. This pulse wave progresses through the vasculature from compliant region to compliant region at a rate dependent upon the resistance and compliance of each region. Limits to flow around the system are produced by the diastolic volume capacity of the ventricles, the flow limitation to venous drainage that occurs when the pressure inside the floppy veins is less than the pressure outside the vessels, and the time limits imposed by time constants of drainage on the movement of the volume wave due to the fixed cycle time determined by heart rate. These mechanical factors can have a much larger impact than actual changes in blood volume. Finally, clinical responses to treatments can only be in the realm of the physiologically possible.
