# Erratum to: Volume and its relationship to cardiac output and venous return

**DOI:** 10.1186/s13054-016-1571-3

**Published:** 2017-01-26

**Authors:** S. Magder

**Affiliations:** Department of Critical Care, McGill University Health Centre, 1001 Decarie Blvd, Montreal, Quebec H4A 3J1 Canada

## Erratum

Unfortunately, the original version of this article [1] contained an error. The legend to Fig. 6 is incorrect. It should read a “A decrease in capacitance is the same as lowering the opening on the side of a tub” instead of “increase”. Please find the correct figure legend below.

**Fig. 6:**
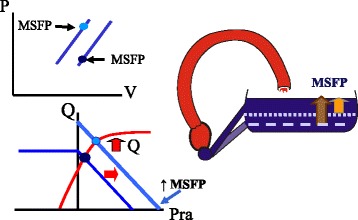
Change in cardiac output and venous return with an increase in capacitance. A decrease in capacitance is the same as lowering the opening on the side of a tub for it allows more volume to flow out, which is the equivalent of more volume being stressed. Graphically it results in a leftward shift of the volume–pressure relationship of the vasculature (*upper left*). This shifts the venous return curve to the right and increases cardiac output through the Starling mechanism (*lower left*). This effect is identical to giving volume to expand stressed volume. *Pra* right atrial pressure

Fig. 6 Change in cardiac output and venous return with an increase in capacitance. A decrease in capacitance is the same as lowering the opening on the side of a tub for it allows more volume to flow out, which is the equivalent of more volume being stressed. Graphically it results in a leftward shift of the volume–pressure relationship of the vasculature (*upper left*). This shifts the venous return curve to the right and increases cardiac output through the Starling mechanism (*lower left*). This effect is identical to giving volume to expand stressed volume. *Pra* right atrial pressure.
